# Development of EST‐SSR markers for *Pluchea indica* (Asteraceae) and cross‐amplification in related species

**DOI:** 10.1002/aps3.1173

**Published:** 2018-08-20

**Authors:** Yi Yang, Sixin Huang, Yanling Wang, Lingjian Gui, Yiran Liu, Xiaomei Huang, Guoqingzi Chen, Fengxiao Tan, Jianwu Wang

**Affiliations:** ^1^ Key Laboratory of Tropical Agro‐Environment Ministry of Agriculture South China Agricultural University Guangzhou 510642 Guangdong People's Republic of China; ^2^ College of Natural Resources and Environment South China Agricultural University Guangzhou 510642 Guangdong People's Republic of China; ^3^ College of Agriculture South China Agricultural University Guangzhou 510642 Guangdong People's Republic of China; ^4^ Guangxi Botanical Garden of Medicinal Plants Nanning 530023 Guangxi People's Republic of China

**Keywords:** Asteraceae, microsatellite marker, *Pluchea indica*, transcriptome

## Abstract

**Premise of the Study:**

Expressed sequence tag–simple sequence repeat (EST‐SSR) markers were developed for *Pluchea indica*, a traditional medicinal species widespread along the tropical coastlines of Asia and northern Australia.

**Methods and Results:**

Based on transcriptome data for *P. indica*, a total of 40 primer pairs were initially designed and tested, of which 17 were successfully amplified and showed clear polymorphism. For these SSR loci, one to nine alleles per locus were identified. The levels of observed and expected heterozygosity ranged from 0 to 0.9 and 0 to 0.831, respectively. Furthermore, 16, 17, and 12 loci were successfully amplified in three congeneric species, *P. eupatorioides*,* P. pteropoda*, and *P. sagittalis*, respectively.

**Conclusions:**

The SSR markers described here may be useful for further investigation of the population genetics of *P. indica* and related species.

*Pluchea* Cass. (Asteraceae: Inuleae) consists of approximately 80 species, some of which can be used as herbal medicines (Sharma and Goyal, 2011). *Pluchea indica* (L.) Less., known as Indian camphorweed or Indian marsh fleabane, is a widely branching shrub species, native to much of Asia and northern Australia (Anderberg, 1994). This species is typically found in coastal areas and occasionally in woodland at low elevations (Peng et al., 1998). Currently, it is also widespread in the Pacific Islands as an introduced and often invasive species (Motooka et al., 2003; PIER, 2012). *Pluchea indica* is a traditional medicine for its antioxidant, anti‐ulcer, antinociceptive, anti‐inflammatory, antimicrobial, anticancer, and anti‐amoebic activities (Choi and Hwang, 2005; Sharma and Goyal, 2011; Cho et al., 2017). The chemical β‐sitosterol extracted from the root of *P. indica* can relieve the toxicity of the venom of Russell's viper and the monocled cobra (Gomes et al., 2007). Thus, it has received increasing attention as a traditional herbal medicine in Asia, especially in China, India, and Southeast Asia. However, most research on *P. indica* to date has focused on phytochemistry and cytology (Cho et al., 2012; Kao et al., 2015). The actual levels of the genetic diversity and gene flow among populations of *P. indica* across its range have never been studied, which limits the survey and utilization of this valuable resource. In this study, we developed a set of novel expressed sequence tag–simple sequence repeat (EST‐SSR) markers for future population genetic studies in *P. indica*. Additionally, we tested the transferability of these markers in three related species (*P. eupatorioides* Kurz, *P. pteropoda* Hemsl., and *P. sagittalis* (Lam.) Cabrera) that are commonly found in southern China.

## METHODS AND RESULTS

Fresh leaves of *P. indica* for total RNA extraction were collected from Wenchang, Hainan, China, and were frozen in liquid nitrogen before storage at −80°C. A modified cetyltrimethylammonium bromide (CTAB) method was used to extract total RNA from the sample (Chen et al., 2011). The mRNA was then isolated using Oligotex‐dT30 (TaKaRa Biotechnology Co., Dalian, Liaoning, China) and fragmented ultrasonically. The cDNA libraries from a single plant were prepared for paired‐end short‐read sequencing following the Illumina protocol. Sequencing was performed on an Illumina HiSeq 2500 sequencing platform (HonorTech Co., Beijing, China). Trinity software (Grabherr et al., 2011) was used with the default parameters to assemble reads, and 22,314,967 paired‐end reads were obtained. A total of 134,512 contigs were obtained, with an average length of 771 bp, an N50 length of 1248, and an average depth of coverage of 32.24×. All sequence information was uploaded onto the National Center for Biotechnology Information (NCBI) Sequence Read Archive (accession no. SRR6650047). MIcroSAtellite identification software (MISA) with default settings (Thiel et al., 2003) was used to detect SSRs. The criteria for identifying SSR motifs were as follows: the minimum number of nucleotide repeats was five for hexanucleotide, pentanucleotide, tetranucleotide, or trinucleotide repeat motifs, and six for dinucleotide repeats. A total of 28,983 SSR regions were found. Primer3 software (Rozen and Skaletsky, 1999) was further used to design primers, with optimum conditions of a length of 20 bp (18–22 bp), an annealing temperature of 60.0°C, and a product size range of 150–300 bp.

Forty SSR loci were initially developed, and 68 samples from three natural populations of *P. indica* were used to evaluate their polymorphic content (Appendix 1). Genomic DNA was isolated from silica‐dried leaves using the HiPure Plant DNA Mini Kit (Magen, Guangzhou, Guangdong, China) following the manufacturer's protocol. PCR amplifications were performed in final volumes of 15 μL, including 15 ng of genomic DNA, 1× PCR buffer (10 mM Tris‐HCl [pH 8.4] and 1.5 mM MgCl_2_; TransGen Biotech Co., Beijing, China), 0.2 mM dNTPs (TransGen Biotech Co.), 0.5 μM of each primer (BGI, Beijing, China), and 0.5 units EasyTaq DNA polymerase (TransGen Biotech Co.). The PCR reactions were implemented on a Bio‐Rad PTC‐200 thermocycler (Bio‐Rad Laboratories, Hercules, California, USA) using the following conditions: initial denaturation at 94°C for 4 min; followed by 31 cycles of 94°C for 1 min, 40 s at the specific annealing temperature (53°C or 56°C, see Table 1), and 75 s at 72°C; with a final extension of 10 min at 72°C. PCR products were detected by electrophoresis through 1% TAE agarose gels. Finally, among the 40 selected primer pairs, 17 were successfully amplified. The successfully amplified SSR loci were sequenced, and the sequences were uploaded onto GenBank (accession no. MG893053–MG893069; Table 1). SSR genotyping was performed on a Fragment Analyzer Automated CE System (Advanced Analytical Technologies [AATI], Ames, Iowa, USA) with the Quant‐iT PicoGreen dsDNA Reagent Kit (the 35–400 bp Range DNA Ladder included; Invitrogen, Carlsbad, California, USA). Allele sizes and the number of alleles per locus were scored using PROSize 2.0 software (AATI) with manual correction. GenAlEx 6.5 software (Peakall and Smouse, 2012) was used to compute the average number of alleles per locus, the observed heterozygosity, and the expected heterozygosity of each SSR locus. Hardy–Weinberg equilibrium (HWE) was tested using GENEPOP 4.3 (Rousset, 2008). The SSR data were also tested for null alleles with MICRO‐CHECKER (van Oosterhout et al., 2004).

**Table 1:** Characteristics of the 17 SSR markers developed for *Pluchea indica*

Locus	Primer sequences (5′–3′)	Repeat motif	*T*_a_ (°C)	*OS* (bp)	GenBank accession no.	Putative function	*E*‐value
PI894	F: CACCTCCACCCTCAATGACT	(GCC)_5_	53	261–270	MG893053	Glycine‐rich cell wall structural protein 1 [*Ceratitis capitata*]	3e‐08
	R: AGCGGTGCTAGTTTGCATCT						
PI509	F: TTGCAACAATCAAACCATCAA	(ACC)_6_	53	263–275	MG893054	Dof zinc finger protein DOF2.4‐like [*Helianthus annuus*]	2e‐88
	R: TGCAAAAGTGTCGTGGTTGT						
PI595	F: AACCTCCACTCGATCATTGC	(TA)_6_	53	180–188	MG893055	Uncharacterized LOC110898652 [*Helianthus annuus*]	7e‐18
	R: CTCTCAGGTACCCACCCAAA						
PI3479	F: ATTGCAGGAGGATGAGGATG	(GAT)_5_	53	257–275	MG893056	Heat shock factor DNA‐binding protein [*Paraphaeosphaeria minitans*]	4e‐05
	R: GATAAGGCGCAAATGGATGT						
PI4937	F: TGGTGGTCCGCCTATCTAAC	(TTC)_5_	53	149–158	MG893057	Proline‐rich receptor‐like protein kinase PERK4 [*Glycine max*]	0.150
	R: TGCAACCACCTCATCACCTA						
PI5055	F: GAGTACACCGCACCAATCCT	(ATC)_6_	53	157–166	MG893058	Uncharacterized protein LOC107469814 [*Arachis duranensis*]	7e‐08
	R: CCTGTGGGGTTGCAGTAGAT						
PI1333	F: TTCCTTCTTTTCTCCATGCAA	(AG)_22_	53	289–297	MG893059	Bidirectional sugar transporter SWEET1‐like [*Nelumbo nucifera*]	3e‐13
	R: TGACTCACCACCCAGTACCA						
PI1151	F: CGGTTTTTGTTTGGGAGAAA	(TGG)_7_	53	218–230	MG893060	GDSL esterase/lipase At4g10955‐like [*Helianthus annuus*]	2e‐115
	R: GCGGCCTAAAATTCTCCTTC						
PI1081	F: TGGCTGTGGTTTTGGTTGTA	(GCG)_5_	53	157–160	MG893061	Protein tesmin/TSO1‐like CXC 5 isoform X2 [*Helianthus annuus*]	3e‐77
	R: TAGTGTTGGTGGCAGTGCAT						
PI1628	F: GGGCTAGCTGGATCTCACTG	(CT)_12_	53	230–262	MG893062	Polygalacturonase QRT3 [*Prunus persica*]	0.024
	R: AGCGAGTGTGAAGCCTCAAT						
PI2036	F: GCTCATTGCAAGGTAGCACA	(TGACGA)_5_	53	152–176	MG893063	Uncharacterized protein LOC107174080 [*Diuraphis noxia*]	6e‐07
	R: GCCAAAATTGATGTTTCATACTTTC						
PI20846	F: TCTTCTCGCCACACACTCTG	(CTTCA)_5_	56	298–318	MG893064	Elongation factor 1‐beta‐like [*Helianthus annuus*]	0.000
	R: CAGCTGCGTAAACCTTCACA						
PI22873	F: TATGGCTGCTGCTGCATTAC	(GGTTCG)_5_	56	211–217	MG893065	Auxin response factor 6‐like isoform X1 [*Helianthus annuus*]	0.000
	R: AAGATGAGGCTGTGGGTTTG						
PI23228	F: ACTTCACACCCGAAGGTCAC	(TTGT)_5_	56	257–273	MG893066	Probable arabinosyltransferase ARAD1 isoform X1 [*Helianthus annuus*]	0.000
	R: TTTGATTCCTCAAAAAGTTCCTG						
PI23317	F: TGCGTCAAAATGTTGTTGGT	(TGA)_6_	53	161–164	MG893067	Protein OVEREXPRESSOR OF CATIONIC PEROXIDASE 3 [*Helianthus annuus*]	0.000
	R: GCAAGTGCTTCCTCCAACTC						
PI24416	F: GCAATTCGAGAAGGATTCCA	(TGA)_6_	56	263–275	MG893068	Protein PLASTID MOVEMENT IMPAIRED 1‐RELATED 1‐like [*Helianthus annuus*]	0.000
	R: CCAAGTGGAGGAAGCTCAAG						
PI25660	F: GCAGGAGAGGCAATCTGTTC	(CAT)_7_	56	257–266	MG893069	Uncharacterized protein ycf45 [*Helianthus annuus*]	0.000
	R: TAATCCCAAAAGGGGAAACC						

Note

*OS* = observed band size; *T*_a_ = annealing temperature.

In *P. indica*, the number of alleles per locus ranged from one to nine with a mean of 2.716, the levels of observed heterozygosity ranged from 0 to 0.9, and the levels of expected heterozygosity ranged from 0 to 0.831 (Table 2). Fifteen out of the 17 polymorphic SSR loci showed significant deviations from HWE in different populations (*P* < 0.001) (Table 2). Those loci without HWE also showed signs of null alleles (null allele frequency >5%), which indicated that deviations from HWE may have been related to the presence of null alleles. The cross‐species transferability and polymorphism of the 17 SSR markers developed for *P. indica* were further tested in three congeneric species (Appendix 1). In *P. eupatorioides*,* P. pteropoda*, and *P. sagittalis*, the numbers of SSR primer pairs that could be successfully amplified were 16, 17, and 12, respectively, and the numbers of polymorphic SSR loci were 14, 14, and 9, respectively (Table 3).

**Table 2:** Characterization of 17 SSR markers in three populations of *Pluchea indica*.^a^

Locus	PIWC population (*N* = 22)			PIHP population (*N* = 23)			PIKCF population (*N* = 23)		
	*A*	*H*_o_	*H*_e_	*A*	*H*_o_	*H*_e_	*A*	*H*_o_	*H*_e_
PI894	3	0.000	0.244^b^	2	0.000	0.287^b^	2	0.000	0.083
PI1628	9	0.136	0.831^b^	6	0.087	0.699^b^	5	0.478	0.749^b^
PI1151	4	0.591	0.557	2	0.000	0.499^b^	2	0.000	0.159
PI3479	6	0.818	0.715^b^	6	0.870	0.733	4	0.130	0.447^b^
PI1081	1	0.000	0.000	2	0.000	0.386^b^	2	0.000	0.083
PI5055	3	0.000	0.566^b^	1	0.000	0.000	2	0.000	0.340^b^
PI2036	3	0.000	0.475^b^	3	0.000	0.363^b^	2	0.000	0.227^b^
PI509	4	0.182	0.600^b^	3	0.000	0.480^b^	3	0.391	0.600
PI4937	3	0.000	0.417^b^	3	0.000	0.234^b^	4	0.304	0.661^b^
PI595	5	0.682	0.619^b^	2	0.000	0.476^b^	4	0.304	0.612^b^
PI1333	4	0.000	0.583^b^	4	0.000	0.427^b^	3	0.000	0.363^b^
PI20846	2	0.136	0.127	2	0.087	0.159	3	0.087	0.299^b^
PI22873	1	0.000	0.000	1	0.000	0.000	2	0.348	0.454
PI23228	4	0.900	0.641^b^	3	0.043	0.124	2	0.043	0.043
PI23317	1	0.000	0.000	1	0.000	0.000	2	0.174	0.159
PI24416	4	0.750	0.731^b^	2	0.000	0.499^b^	2	0.000	0.386^b^
PI25660	3	0.000	0.177^b^	2	0.000	0.083	2	0.000	0.087

Note

*A* = number of alleles; *H*_e_ = expected heterozygosity; *H*_o_ = observed heterozygosity; *N* = number of individuals analyzed.

a Locality and voucher information are provided in Appendix 1.

b Significant deviation from Hardy–Weinberg equilibrium after sequential Bonferroni corrections (*P* < 0.001).

**Table 3:** Characterization of 17 SSR markers developed for *Pluchea indica* in three closely related species.^a^

Locus	*P. pteropoda* (*N* = 17)			*P. sagittalis* (*N* = 14)			*P. eupatorioides* (*N* = 17)		
	*A*	*H*_o_	*H*_e_	*A*	*H*_o_	*H*_e_	*A*	*H*_o_	*H*_e_
PI894	2	0.000	0.111	—	—	—	4	0.941	0.685
PI1628	4	0.059	0.621	—	—	—	4	0.059	0.569
PI1151	3	1.000	0.614	1	0.000	0.000	2	0.000	0.208
PI3479	4	1.000	0.666	3	0.000	0.561	3	0.000	0.443
PI1081	4	0.000	0.657	3	0.071	0.584	3	0.000	0.457
PI5055	3	1.000	0.623	2	0.000	0.245	2	0.000	0.484
PI2036	1	0.000	0.000	1	0.000	0.000	2	1.000	0.500
PI509	3	0.706	0.493	4	0.857	0.666	—	—	—
PI4937	3	0.294	0.569	2	0.000	0.337	4	0.647	0.673
PI595	5	1.000	0.663	—	—	—	6	0.588	0.689
PI1333	3	0.059	0.258	—	—	—	4	1.000	0.625
PI20846	2	0.000	0.360	—	—	—	3	0.000	0.215
PI22873	1	0.000	0.000	1	0.000	0.000	1	0.000	0.000
PI23228	5	0.824	0.696	3	0.071	0.446	1	0.000	0.000
PI23317	1	0.000	0.000	3	0.143	0.135	3	0.000	0.304
PI24416	4	0.941	0.580	2	0.286	0.245	3	0.118	0.388
PI25660	2	0.000	0.219	4	0.071	0.503	2	0.000	0.457

Note

— = not available; *A =* number of alleles; *H*_e_ *=* expected heterozygosity; *H*_o_ *=* observed heterozygosity; *N* = number of individuals analyzed.

a Locality and voucher information are provided in Appendix 1.

## CONCLUSIONS

This is the first set of SSR markers developed for the genus *Pluchea*. The 17 SSR loci reported herein are efficient in studying the genetic variation and population structure of *P. indica*. We are currently using these SSR loci to evaluate the dispersal distances of *P. indica* seeds along tropical coasts of the Indo‐West Pacific region, especially of South China, and we have found great divergence between *P. indica* populations from the South China coasts and those from other areas of the Indo‐West Pacific region (F. Tan, Y. Yang, S. Huang, Y. Wang, X. Huang, Y. Huang, and J. Wang, unpublished data). Furthermore, the successful transferability of these markers to congeneric species suggests that they may be useful in studies of related species in *Pluchea*.

## DATA ACCESSIBILITY

All sequence information was uploaded to the National Center for Biotechnology Information (NCBI) Sequence Read Archive (accession no. SRR6650047); primer sequences were uploaded to GenBank (accession no. MG893053–MG893069; Table 1).

## APPENDIX 1 Sampling information for species in this study. All voucher specimens are deposited at the herbarium of Sun Yat‐sen University (SYS), Guangzhou, China.

**Table:**

Species	Population code	Voucher no.	Collection locality	Geographic coordinates	*N*
*Pluchea indica* (L.) Less.	PIWC	Huang121117	Wenchang, Hainan, China	19°37′42.24″N, 110°47′37.81″E	22
	PIHP	Huang130326	Beihai, Guangxi, China	21°36′16.30″N, 109°41′42.79″E	23
	PIKCF	Huang131120	Kuching, Malaysia	1°40′12.12″N, 110°19′44.00″E	23
*P. eupatorioides* Kurz	PEC	Zeng171204	Nanning, Guangxi, China	22°53′11.92″N, 108°14′31.36″E	17
*P. pteropoda* Hemsl.	PPQYA	Huang171101	Danzhou, Hainan, China	19°44′17.21″N, 109°13′4.88″E	17
*P. sagittalis* (Lam.) Cabrera	PSZH	Shixg171202	Zhuhai, Guangdong, China	22°16′8.01″N, 113°35′8.48″E	14

Note

*N* = number of individuals analyzed.
